# Artificial Tongue Embedded with Conceptual Receptor for Rubber Gustatory Sensor by Electrolytic Polymerization Technique with Utilizing Hybrid Fluid (HF)

**DOI:** 10.3390/s22186979

**Published:** 2022-09-15

**Authors:** Kunio Shimada

**Affiliations:** Faculty of Symbiotic Systems Sciences, Fukushima University, Fukushima 960-1296, Japan; shimadakun@sss.fukushima-u.ac.jp; Tel.: +81-24-548-5214

**Keywords:** gustation, gustatory receptors, mimesis, tongue, rubber, electrolytic polymerization, hybrid fluid (HF), robotics, smart material

## Abstract

The development of gustatory sensors is essential for the development of smart materials for use in robotics, and in the food, beverage, and pharmaceutical industries. We therefore designed a prototype of a rubber tongue embedded with a gustatory receptor mimicking a human tongue using our previously proposed hybrid fluid rubber (HF rubber) and an electrolytic polymerization technique. The fabricated gustatory receptor was composed of Pacinian corpuscles, which are well known and have already been elucidated as effective haptic and auditory receptors in previous studies. Moreover, the receptor has self-powered voltage generated as built-in electricity as a result of the ionized particles and molecules in the HF rubber. The utilization of a layered structure for the Pacinian corpuscles induced a typical response not only to normal and shear forces but to thermal variations. Typical gustatory characteristics, including the initial response voltage and the cyclic voltammogram form, were clearly varied by five tastes: saltiness, sourness, sweetness, bitterness, and umami. These results were due to ORP, pH, and conductivity.

## 1. Introduction

Myriad fields such as automated logistics and supply chain systems, digital manufacturing, robotics, autonomous vehicles and machines, etc., require sensors to aid their performance. This may involve electric signals reacting to an amalgam of temperature, motion, posture, and chemical or ionic conditions, among others [1,2]. Autonomic sensing systems may meet the necessary requirements and work with wearable systems. Wearable sensor technology is also becoming the focus of intense activity in scientific fields [3]. The performance of sensing systems comprises sensors and computational networks to control the electric signals obtained from the sensor. The sensor can be used to simulate the five senses: gustation, olfaction, auditory sensation, tactile sensation, and vision; meanwhile, the computational network simulates the functions of the nerves and brain. As examples of engineering applications, gustatory sensors are applicable as tasting sensors in pharmaceutical manufacturing and food industries, where the quality of beverages, foods, etc., must be monitored [4]. Olfactory sensors are essential for quality control of volatile substances and aromas in cosmetics, environmental control in manufacturing industries, and so on [5]. Auditory sensors are important for the auditory systems of robots that must listen to the user’s instructions [6,7], while tactile sensors are useful in diverse devices to keep track of variable conditions of temperature, vibration, softness, force, etc. [5]. Investigations into the fabrication and configuration of these sensors are therefore significant for facilitating many engineering applications.

Regarding tactile sensation, the premier artificial soft skin is currently electronic skin (i.e., e-skin) [8,9], which is integrated with elastomeric substrates and sensory-induced fillers using a chemical process. The mechanisms by which pressure or force induces a response to a haptic sensation are based on several physical sensing mechanisms: piezo-resistivity, piezo-electricity, piezo-capacitivity, and tribo-electricity [10]. It is possible to develop a configuration of human sensory receptors as state-of the-art biological mimicry by ensuring the receptor is induced by the several physical sensing mechanisms. Human sensory receptors as mechanical or thermal receptors are classified: nerve endings, Meissner corpuscles, Pacinian corpuscles, Merkel cells, and Ruffini endings. E-skin as a tactile sensor mimicking the receptors is one configuration of artificial skin [11,12]. One of its merits is self-powered electric signals for responding to extraneous force and temperature, whose mechanisms derive from the behavior of ionic particles and molecules in the material of the artificial skin, inducing the piezo-electricity, piezo-capacitivity, and tribo-electricity. Meanwhile, in previous reports, gustatory sensors have mimicked the mechanism of the brain affected by gustatory receptors [13], with the artificial tongue sensing via gustatory receptors [14,15]. The reaction of such sensors to flavors [16] including sweetness [17], saltiness, sourness [18], bitterness, and umami [19] has been addressed. Artificial olfactory sensors have also been investigated, and their properties clarified [20,21].

The mimicry of the sensory receptors can facilitate their development. Brilliant improvements in the production of rubber-based soft materials require fewer procedural steps. However, production of the sensors that mimic the receptors may be complicated or bulky because the electrical mechanisms of ionic behavior are complex, and have not yet been elucidated well. Therefore, we first attempted to solve the problem by proposing a rubber-based production technique, adopting electrolytic polymerization and a magnetic responsive fluid, hybrid fluid (HF). The electrolytic polymerization enables not only the solidification of rubber but also adhesion between the rubber and the metal wire that provides the sensor’s output. We previously demonstrated the viability of fabricating tactile, equilibrium and auditory sensors with sensory receptors that mimicked the tactile, equilibrium and auditory cutaneous receptors, respectively [22,23]. However, we have not yet investigated gustatory sensors. In the present study, we attempt to elucidate its feasibility. We demonstrate the fabrication of an artificial tongue with gustatory receptors using our proposed rubber-based production techniques. The built-in voltage of the artificial tongue generated by ionic and electro transmission as piezo-electricity, piezo-capacitivity, and tribo-electricity, is measured and compared with the cyclic voltammetric results under five flavors. In addition, we discuss the mechanical and thermal properties of the artificial tongue. Thus, the feasibility of the rubber-made gustatory receptor is demonstrated in the same manner as the auditory and tactile sensors.

## 2. Material

### 2.1. Conceptual Receptor

**Figure 1:**
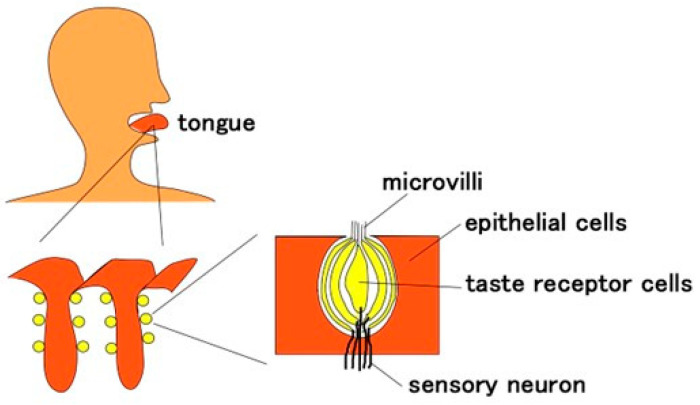
Schematic anatomical diagram of gustatory receptor in a human tongue.

**Figure 2:**
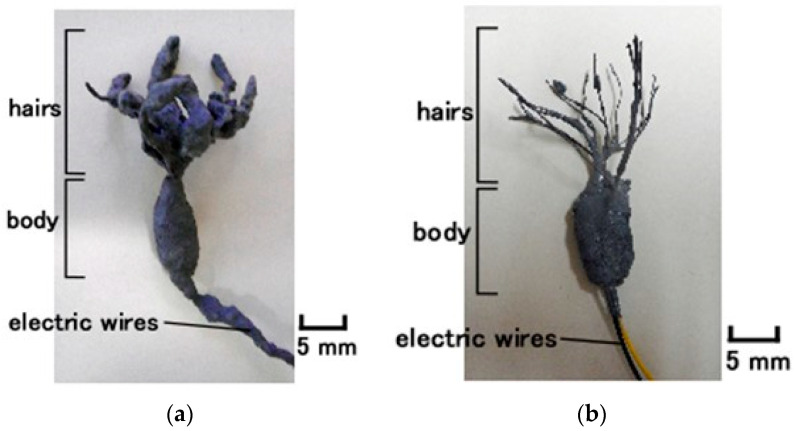
Images of the designed gustatory receptors produced using electrolytic polymerization with HF rubber: (**a**) cylindrical Pacinian corpuscles; and (**b**) layered Pacinian corpuscles.

The tongue consists of perception units of taste bud clusters with taste cells, sensory neurons, etc., as shown in Figure 1. The taste bud as a gustatory receptor comprises plural taste cells reactive to the ionic liquid or molecules and the nerve transmitting the electric signal of the reaction. Taste cells are structured with the gustatory element as a chemical or ionic response, and the nerve as the electric wire transmitting the output sensory signal. The tip of the aggregated taste cells has micro hairs called microvilli. Each taste cell is categorized by flavor, and their structures should be different. For example, the taste cells for sourness and saltiness have an ionically responsive configuration, while those for sweetness, umami, and bitterness have an organically responsive configuration through which G protein is transmitted. In the present study, we mimic the configuration with a two-part ionically responsive body having electric wires and hairs as shown in Figure 2. The body corresponds to the taste receptor cells, and the hairs to the microvilli. Our mimicking body is irrelevant to the difference in the structure for various flavors.

Actual human receptors have several types of mechanical or thermal receptors: nerve endings, Meissner corpuscles, Krause end bulbs, Pacinian corpuscles, Merkel cells, and Ruffini endings. Each receptor responds to a particular sensation—haptic, thermal, vibrating, gustatory, or olfactory. In the present study we adopt the Pacinian corpuscles from a similarly structured configuration by comparison with the actual human gustatory receptor.

### 2.2. HF Rubber

While Shimada has proposed and continues to investigate rubber-made soft artificial skin called hybrid skin (H-skin) [24], magnetic compound fluid (MCF) combined with rubber has been utilized to simulate human cutaneous receptors. MCF is a magnetically responsive intelligent colloidal fluid that consists of 1-μm-diameter ordered metal particles such as Ni, and 10-nm-diameter sphere magnetite particles (Fe_3_O_4_) coated with an oleic acid surfactant in a solvent such as water and kerosene. When a magnetic field is applied, many needle-like or rug-like magnetic clusters are created in the MCF such that they enhance its electrical and thermal conductivity. Rubbers such as natural rubber (NR) and chloroprene rubber (CR) compounded by MCF become conductive rubber. Furthermore, MCF rubber can be mixed simultaneously with diene rubber such as NR and CR, and non-diene rubber such as silicone (Q) and urethane (U) by compounding polyvinyl alcohol (PVA). This is because PVA produces emulsion polymerization between the diene and non-diene rubbers. By electrolytic polymerization, the MCF rubber is solidified without the vulcanization process by sulfur. As the constituents of the HF are water, kerosene, Q, PVA, surfactant, metal and Fe_3_O_4_ particles, which can be compounded by emulsion polymerization, HF can be produced without using a magnetic fluid (MF) [25]. It has the same constituents as MCF; thus, MF is compounded. HF is soluble in water, kerosene and Q. In addition, in contrast to MCF, HF makes the production tractable. It is an advantage that HF does not require MF, as MF is currently becoming unavailable. Incidentally, the kerosene involved in the HF is ordinary oil highly purified up to more than 99.9% which is well known and used experimentally as purified oil in chemistry and material sciences. By the utilization of the solvent of the kerosene as well as other solvents of water and silicon oil (Q-rubber) in the HF, the variegated rubbers such as diene and non-diene rubbers can be soluble simultaneously in the magnetic responsive fluid. The ability of combining these discrepant solvents is achieved by using PVA because PVA can combine these solvents by the emulsion polymerization. The purpose of the development of the HF is to utilize the combination of the variegated discrepant rubbers such as NR, CR, Q, U, and so on, for convenient production of a state-of-the art rubber, which brings about the enhancement of the combined rubber’s property such as the conductivity and piezoelectricity for haptics, various sensations, and so on.

We prepared HF rubber in advance. The components of HF were as follows: 3 g water, 3 g kerosene, 3 g silicon oil (KF96 with 1 cSt viscosity, which would solidify Q; Shin-Etsu Chemical Co., Ltd., Tokyo, Japan), 21 g PVA, 3 g Fe_3_O_4_ particle (Fujifilm Wako Chemicals Co., Ltd., Osaka, Japan), 3 g Fe particles (M300, about 50 μm particles; Kyowa Pure Chemical Co., Ltd., Tokyo, Japan), and 4 g sodium hexadecyl sulfate aqueous solution. All ingredients were mixed in an agitator under air evacuation. After mixing the HF and other ingredients listed in Table 1, HF rubbers 1–4 were electrolytically polymerized. Here, the carbonyl Ni powder had particle sizes on the order of microns, with bumps on the surface. The electric conditions of electrolytic polymerization were the same as those detailed in our previous study on an HF-made artificial auditory receptor [23].

### 2.3. Artificial Receptor and Tongue

**Figure 3:**
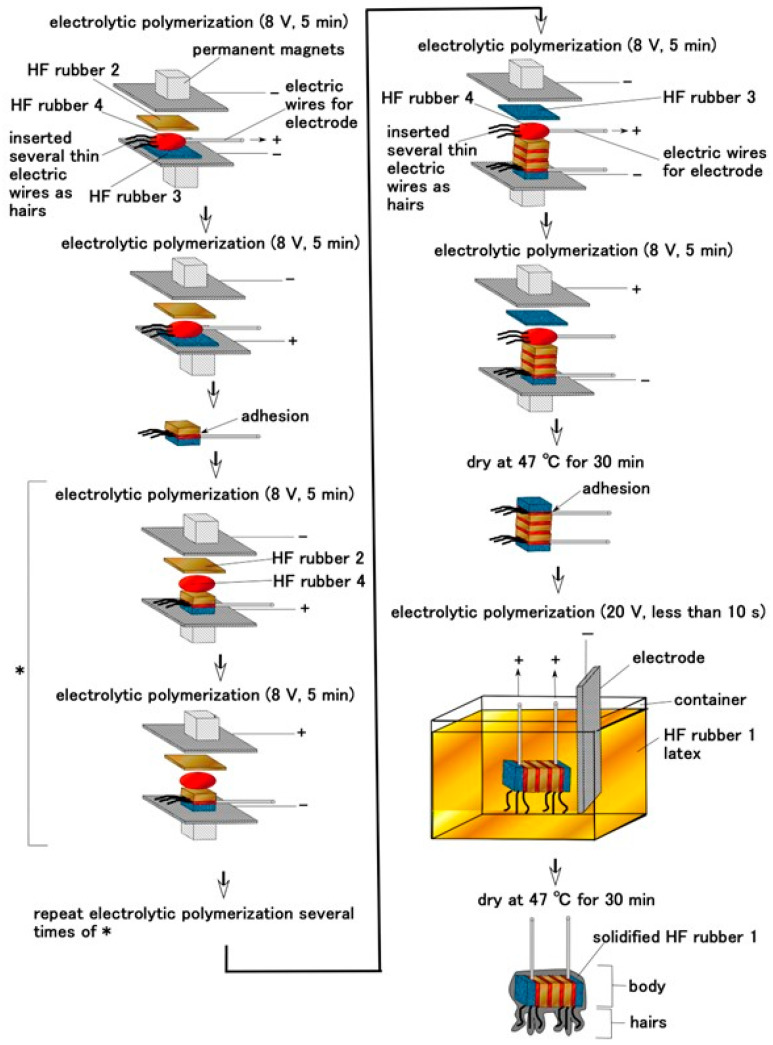
Fabrication process of layered-type Pacinian corpuscles for gustatory receptor.

**Figure A1:**
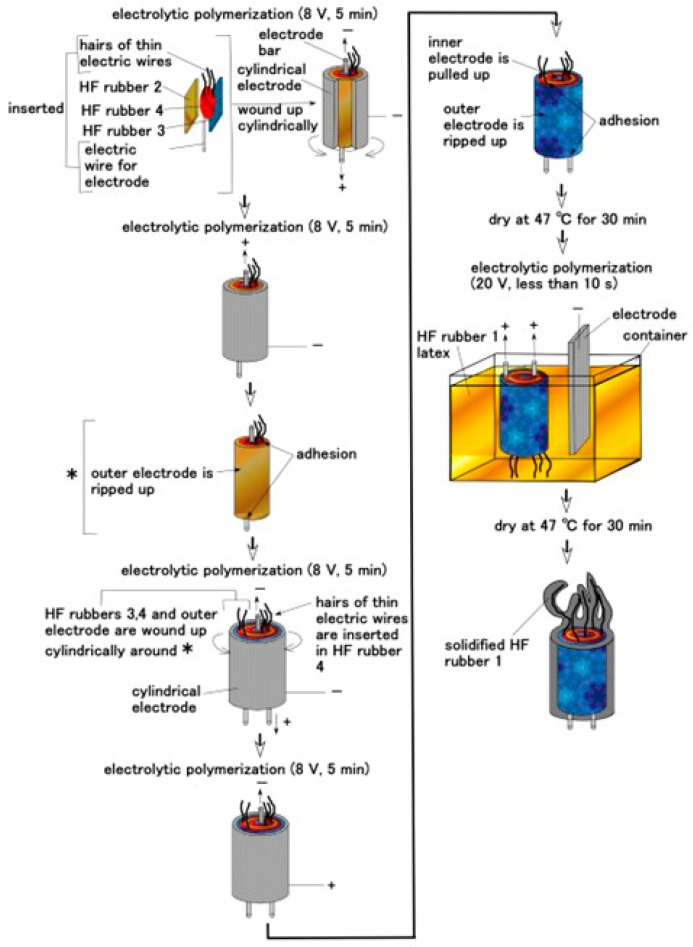
Fabrication process of cylindrical-type Pacinian corpuscles for gustatory receptor [23].

Once HF rubbers 1–4 were prepared, the next phases of fabricating the artificial gustatory receptor and tongue involved the production of the Pacinian corpuscles and the tongue embedded with them. The tongue configuration had layered and cylindrical condensers, and the artificial receptors were simulated by the electronic element. There were predominantly two types of condenser: layered-type and concentric-cylinder condensers. Figure 3 shows the fabrication procedure for the layered type; Figure A1 in the Appendix A illustrates the cylindrical type. The latter was described in our previous study with a detailed explanation of the production conditions [23], similar to the layered type. Therefore, we present here the configuration of the layered type.

The condenser corresponds to the body of the artificial receptor, as shown in Figure 2. The hairs adhered to the body; thin silver-gilt electric wires approximately 0.1 mm in diameter, were inserted in the condenser as shown in Figure 3 and Figure A1. Here we could produce two types of hairs, conductive and non-conductive. For the former, the thin wires were simultaneously connected to the electric pole of wires for the electrode of the receptor so that those electric poles were the same at the time of electrolytic polymerization. As a result, the hairs were electrically conducted to HF rubber 4 and the electric wire for electrode of the receptor. For the non-conductive hairs, the thin wires were not connected to the electric pole of the electric wires for the receptor electrode and were neutralized at the time of electrolytic polymerization.

**Figure 4:**
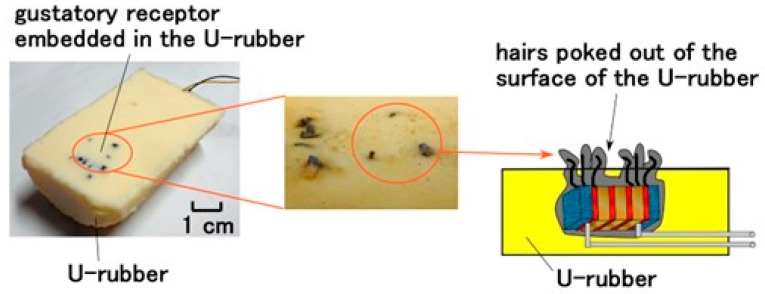
Images and configuration of the designed artificial tongue embedded with gustatory receptor.

As the gustatory receptors are actually embedded in epithelial cells, the simulated receptors were embedded in a soft and elastic rubber made of U-rubber (Human skin gel, 0-solidity; Exseal Co., Ltd., Gihu, Japan) with 1.875 × 10^−6^ Pa^−1^ compressibility so that the artificial tongue could be produced as shown in Figure 4. The hairs poked out of the surface of the U-rubber.

### 2.4. Equivalent Electric Circuit

The Fe_3_O_4_, Fe, Ni, TiO_2_ particles and molecules of rubber, water, and surfactant were ionized by the electrolytic polymerization of HF rubber, such that the rubber served the role of p-type and n-type semiconductors with the configuration of ionized acceptor A^−^ (acceptor) and D^+^ (donor) [23]. The electrons and holes induced from the A and D are mobile, and in the area between them, the electrical situation is neutralized such that there is the formation of a depletion layer. On the other hand, the electrical situation of A^−^ and D^+^ (i.e., the ionized particles and molecules), which is in a static state, becomes the built-in voltage. Consequently, piezo-electricity is generated similar to a condenser. Incidentally, the HF rubber can have simultaneously every characteristic of piezo-resistivity, piezo-capacitivity, and tribo-electricity as well as piezo-electricity. Because of the existence of the electron except for A^−^ and D^+^, it becomes piezo-resistivity. Because of the electric charge among the particles and molecules, it also becomes piezo-capacitivity such as solar cell. Because of the motion of the particles and molecules by deformation, it also becomes tribo-electricity.

**Figure 5:**
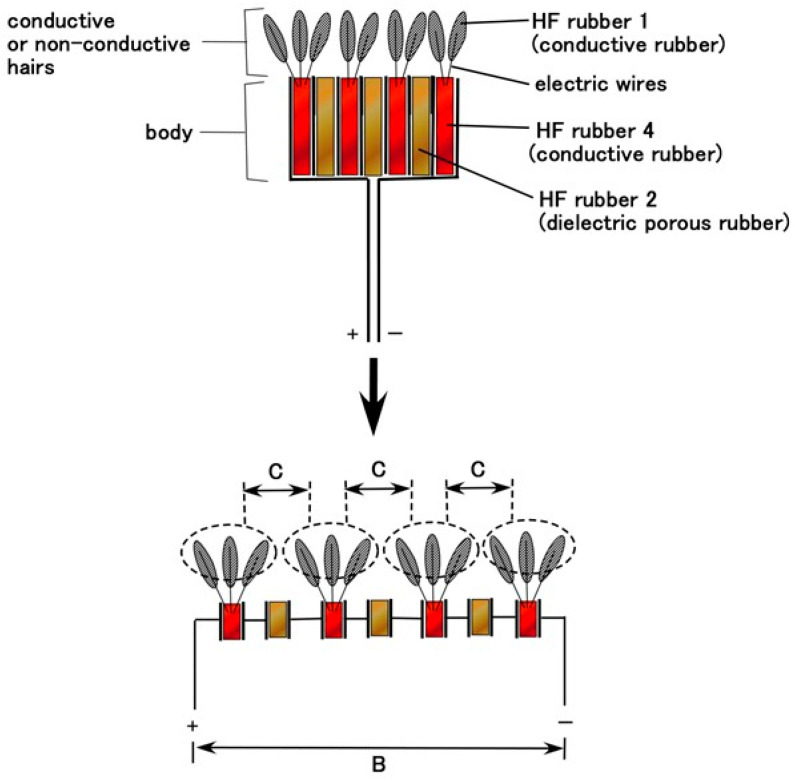
Schematic diagrams of equivalent electric circuit of layered-type Pacinian corpuscles for gustatory receptor.

**Figure 6:**
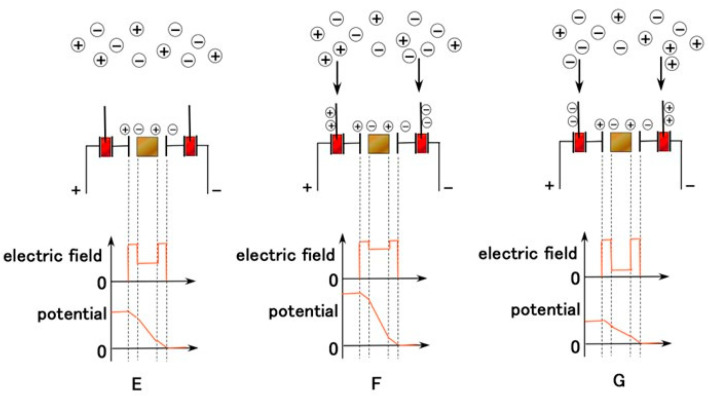
Physical model of electric field and potential in the equivalent electric circuit of Pacinian corpuscles.

**Figure A2:**
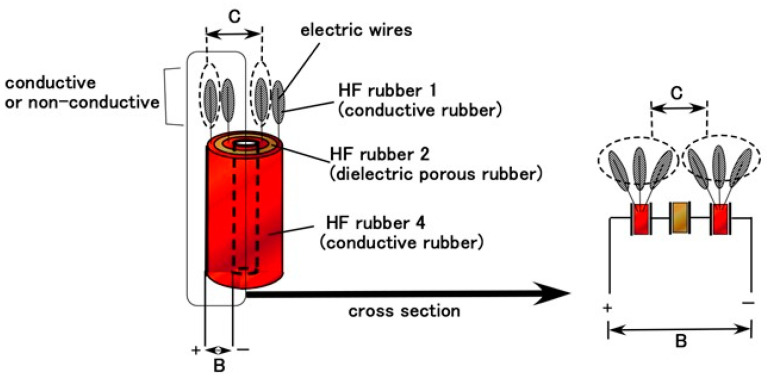
Schematic diagrams of equivalent electric circuit of cylindrical-type Pacinian corpuscles for gustatory receptor [23].

The equivalent electric circuit of the artificial receptor is shown in Figure 5 (layered type) and Figure A2 (cylindrical type). The difference between them is that of condenser shape, layered and concentric-cylindrical shaped condensers, respectively. The built-in voltage is evaluated as B in the figures. The electrical behavior of C affects the built-in voltage because the extraneous ions of other particles and molecules around the thin wires charge electrically to the thin wires, as shown in Figure 6. Contrast this to the situation in which the extraneous ions do not charge on the thin wires (E in Figure 6). The potential between the condenser ultimately increases in the case of thin-wire enhancement by inducement from the extraneous ions (F in Figure 6), and decreases in case of thin-wires diminishing by induction from the extraneous ions (G in Figure 6). The potential is evaluated by the built-in voltage. The potential is quantitatively different depending on the shape of the layered and concentric-cylindrical condensers. In addition, the vibration or motion of the thin wires in the hairs induced by extraneous movement brings about changes in the built-in voltage.

## 3. Experimental Procedure

### 3.1. Mechanical and Thermal Response

Contrary to the actual behavior of the human tongue, the artificial tongue is supposed to be movable mechanically as well as responsive gustatorily to a food or beverage at a certain temperature. We investigated tactile sensing on solids and liquids with various thermal values, as well as gustation.

**Figure 7:**
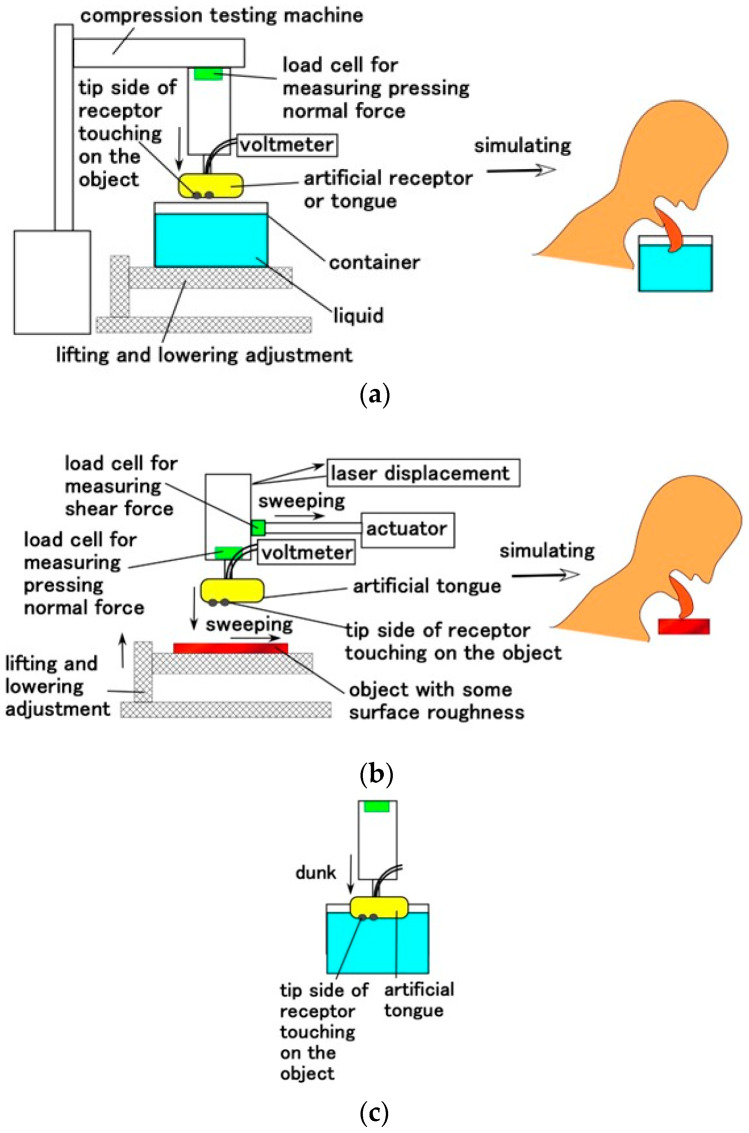
Schematic diagram of the experimental apparatus: (**a**) for the normal force experiment simulating dipping the tongue into a liquid for thermal and gustatory response and a rigid body for tactile response; (**b**) for the shear force experiment simulating licking a rigid body for tactile response; and (**c**) for the touching experiment of thermal and gustatory substances.

For tactile sensing, we adopted the same experimental apparatus used in our previous study to investigate the dynamic properties of normal and shear forces [23] (Figure 7). The application of the normal force corresponds to the situation in which the tongue touches a solid or liquid body. The present tactile experiment with normal force used a solid body, and the thermal experiment was conducted using water. The up and down motion of the artificial tongue attached to the testing machine was repeated five times using a tensile and compressive machine (SL-6002; IMADA-SS Co., Ltd., Toyohasi, Japan) at a velocity of 50 mm/min for a rigid plate without a container, and 100 mm/min for a liquid in a container (Figure 7a). On the other hand, the application of the shear force corresponds to the situation in which the tongue touches a solid or liquid body. The present tactile experiment with shear force used a solid body. The tongue was moved to contact an object with a surface roughness using an actuator with 50 mm/min sweeping speed and approximately 0.02 N normal force (Figure 7b). Two types of objects were used: the first was composed of several aligned sandpapers with different surface roughnesses at equal intervals on a sleek acrylic resin surface. Surface roughness was #180 (*R_a_* = 14.09 μm, *R_q_* = 17.0 μm, *R_y_* = 66.2 μm), #150 (*R_a_* = 14.14 μm, *R_q_* = 17.88 μm, *R_y_* = 81.7 μm), and #100 (*R_a_* = 16.01 μm, *R_q_* = 19.56 μm, *R_y_* = 84.5 μm) on a smooth acrylic resin surface with *R_a_* = 0.03 μm, *R_q_* = 0.03 μm, *R_y_* = 0.2 μm. The second object was created from several pairs of concave and convex bodies, 3 mm in height and 4 mm in width of gutter.

### 3.2. Gustatory Response

**Figure 8:**
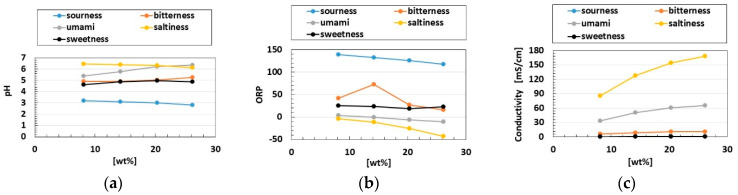
Properties of the substances used for the five tastes: (**a**) pH; (**b**) ORP; (**c**) conductivity, the line of sweetness taste lies on top of the one of sourness one another because of sourness’s diminutive quantitative values.

To examine gustatory sensing, we adopted five tastes: sweetness (a sugar solution in water); saltiness (a salt solution in water); sourness (a rice vinegar solution in water), bitterness (a familiar coffee solution in water); and umami (a favorite Japanese-food tuna powder solution in water). These liquids were poured into a container (Figure 7a). The liquids had redox potential (ORP), pH, and conductivity values (Figure 8). The first was measured with an ORP instrument (YK-23RP-ADV, SatoTech Co., Ltd., Kanagawa, Japan), the second with a pH meter (PH-208, SatoTech Co., Ltd., Kanagawa, Japan), and the third with an electric conductivity meter (DS-72, LAQUA, Horiba Advanced Techno, Co., Ltd., Kyoto, Japan). The artificial tongue touched the liquid at a velocity of 100 mm/min (Figure 7c).

## 4. Results and Discussion

### 4.1. Mechanical and Thermal Response

**Figure 9:**
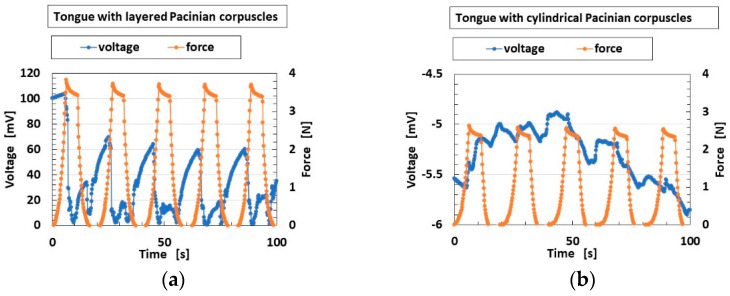
Experimental results of built-in voltage by normal force: (**a**) for layered Pacinian corpuscles; and (**b**) for cylindrical Pacinian corpuscles.

**Figure 10:**
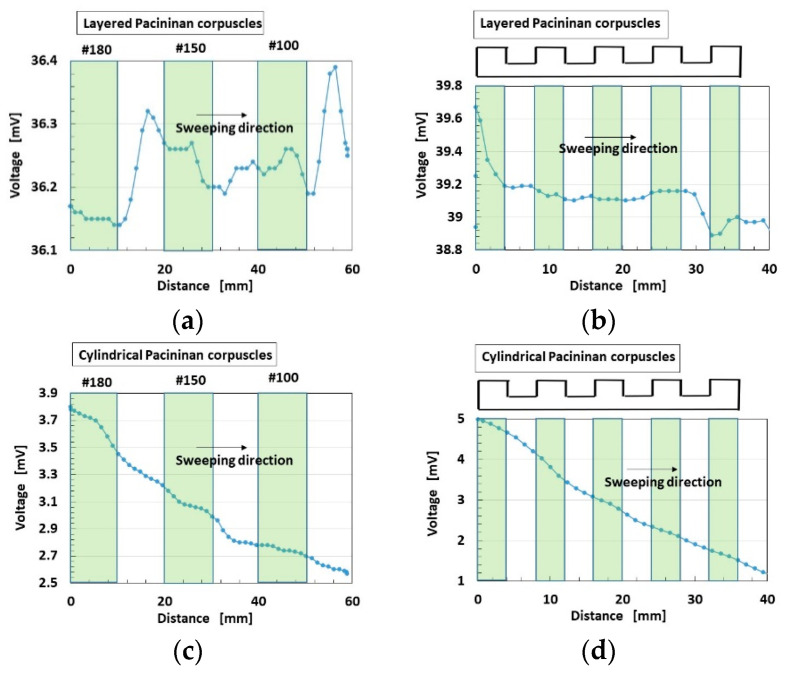
Experimental results of built-in voltage by shear force: (**a**,**b**) for layered Pacinian corpuscles; (**c**,**d**) for cylindrical Pacinian corpuscles; (**a**,**c**) on intermittently positioned sandpaper; (**b**,**d**) on a convex/concave-shaped body.

**Figure 11:**
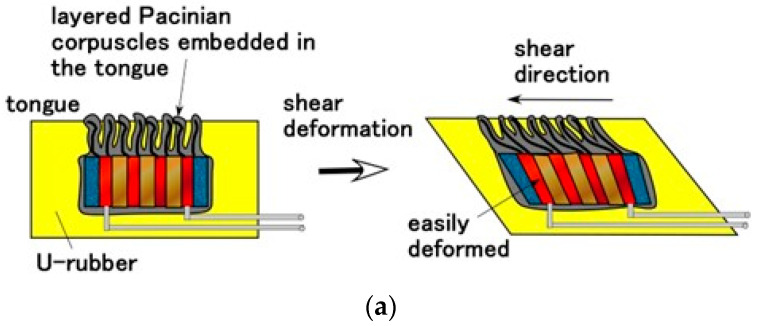

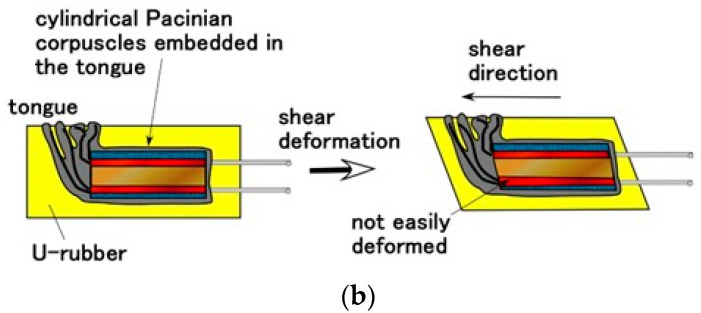
Physical model of results of Figure 10: (**a**) for layered Pacinian corpuscles; (**b**) for cylindrical Pacinian corpuscles.

The changes in built-in voltage by the application of normal force are shown in Figure 9, and by shear force in Figure 10. The layered Pacinian corpuscles had a distinctive response to normal and shear forces compared to the cylindrical type. Regarding normal force, the number of the parts of condenser in the case of the layered type is more than that in the case of the cylindrical type, as compared between Figure 5 and Figure A2. Therefore, the sum of change in the voltage by their deformations in the case of the layered type is more than that in the case of the cylindrical type. As a result, the response to the normal force in the case of the layered type is more than that in the case of the cylindrical type. Regarding shear force, the configuration in the case of the layered type can be deformed by shear motion more easily than that in the case of the cylindrical type, as shown in Figure 11. The HF-rubber receptor embedded in U-rubber is configurated longitudinally along the tongue as shown in Figure 4 and Figure 11. The deformation of the perpendicular multi layers is larger than the axial one of the long cylinder by compression or extension. As a result, the response to the shear force in the case of the layered type is more than that in the case of the cylindrical type.

The results indicate that the layered type had a typical response to the application of force. A few other studies have considered the dynamic response of an artificial tongue made of soft gel regarding the application of normal force [26]. However, this is the first to investigate the application of shear force to an artificial tongue embedded with a receptor. Therefore, our results are of novel value in this field.

**Figure 12:**
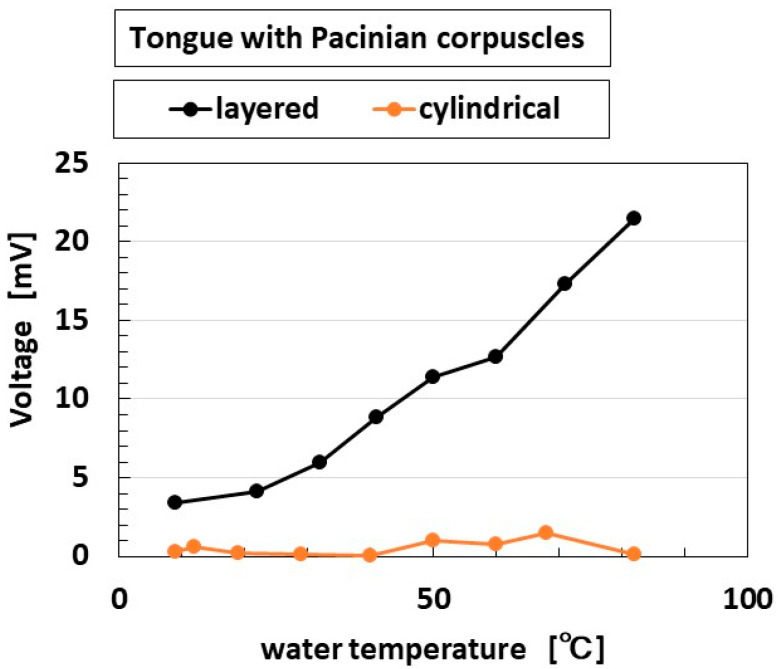
Experimental results of built-in voltage response to thermal variations of water.

Figure 12 shows the thermal response, which corresponds to the tongue touching the surface of cold or hot water (Figure 7c). The thermal case produced similar results to the force response in that the layered type had the typical response to the application of force.

### 4.2. Gustatory Response

**Figure 13:**
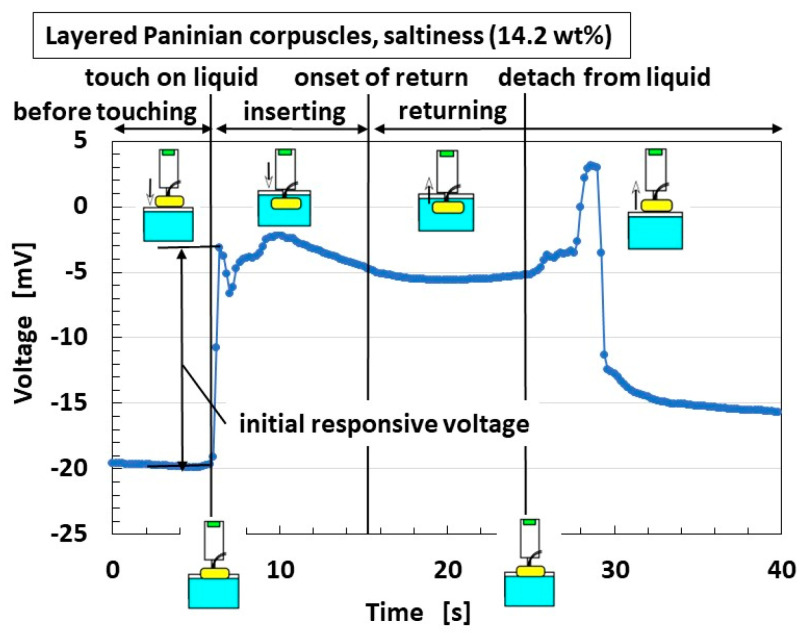
Typical result of built-in voltage responsive to flavored liquid.

Figure 13 presents an example of the changes in the built-in voltage from the artificial tongue. In the simulated situation, the tongue touches a salty liquid, as shown in Figure 7c. The quantitative tendency of changes in the built-in voltage provides the same results as by other various experimental conditions. The voltage changes when the artificial tongue touches the liquid surface, and the difference between that voltage and the initial one before touching is called the “initial response voltage”. We use this parameter in the following figures. This high-enhancement response was repeated when the tongue was removed from the liquid.

**Figure 14:**
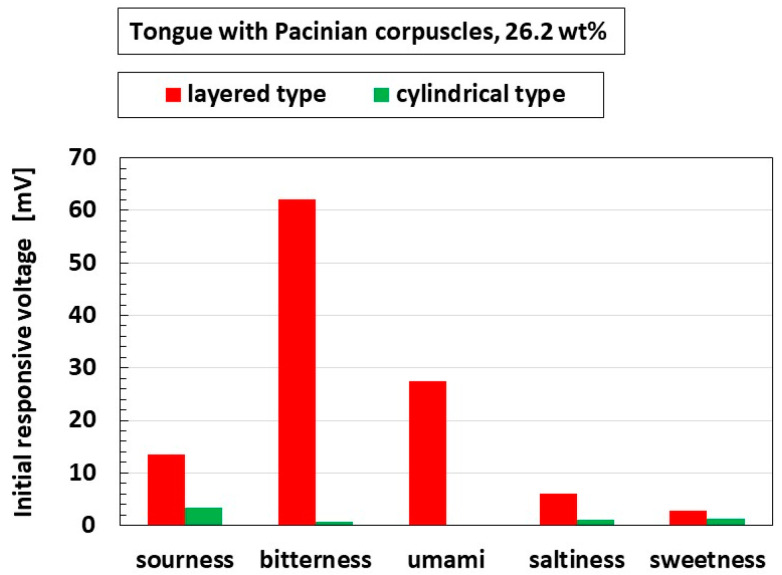
Comparison of initial response voltage between layered and cylindrical Pacinian corpuscles for five tastes.

Figure 14 shows the difference in the initial response voltage between layered and cylindrical Pacinian corpuscles. The gustatory experiment provided the same results as the force and thermal responses in that the layered-type response was larger.

**Figure 15:**
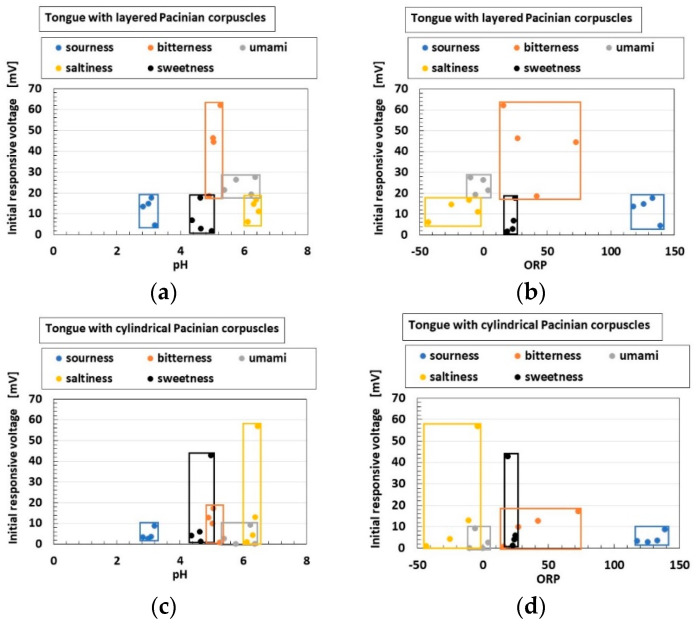
Experimental results of initial response voltage to five tastes: (**a**,**b**) for layered Pacinian corpuscles; (**c**,**d**) for cylindrical Pacinian corpuscles; (**a**,**c**) by pH; (**b**,**d**) by ORP.

Next, we investigated the effects of the five tastes, ORP and pH on the initial response voltage, as shown in Figure 15. The rectangles denote the scope of our using a wide mass concentration of the liquid. In general, the voltage response to the five tastes depended on ORP, pH [13] and the concentration [27] of the liquid, because the gustatory receptors can be categorized into two cases: the receptor responds to the gustation through G protein, or the receptor responds to the gustation by the ions of the liquid percolating through. The former is relevant to bitterness, umami and sweetness, and the latter to sourness and saltiness. In particular, sourness causes H^+^ to percolate, and saltiness Na^+^. In terms of other electrochemical responses, the ion involved in the liquid affecting the receptor cells must be responsive to the taste [17]. Consequently, ORP and pH are significant indicators of the response to the five tastes. We arrange the results by ORP and pH as follows.

In general, for ORP, minus ORP means that the quantity of reductant [red] is larger than that of oxidizer [ox] ([ox] < [red] for ORP < 0, and vice versa [ox] > [red] for ORP > 0); [red] means that electrons are obtained, and [ox] that they are emitted. The transmitting electron produces the gustatory response. Regarding pH, pH > 7 indicates that the quantity of ionized hydrogen [H^+^] is lower than that of the hydroxide ion [OH^−^] ([H^+^] < [OH^−^] for pH > 7, and vice versa [H^+^] > [OH^−^] for pH < 7). For example, the umami-liquid has more [OH^−^] and the sweetness-liquid more [H^+^]. Thus, according to the relations of [ox] and [red], [OH^−^] and [H^+^], respectively, the gustatory response changes. These ions affect the receptor such that the built-in voltage changes by the five tastes (Figure 6). Based on these electrochemical characteristics, the response of the present Pacinian corpuscles varied with the five tastes, as shown Figure 15. The gustatory response differed between the layered and cylindrical configurations.

**Figure 16:**
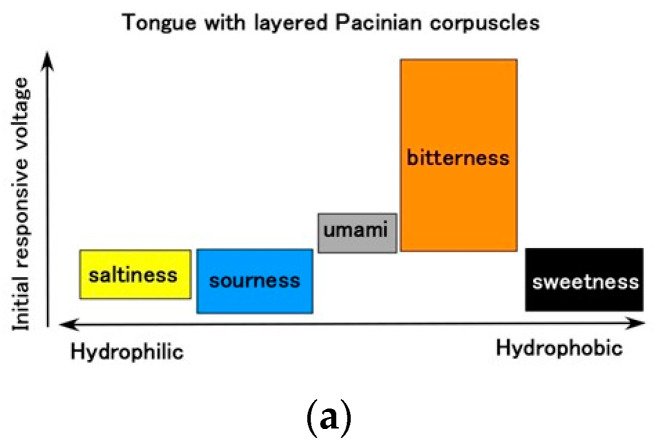

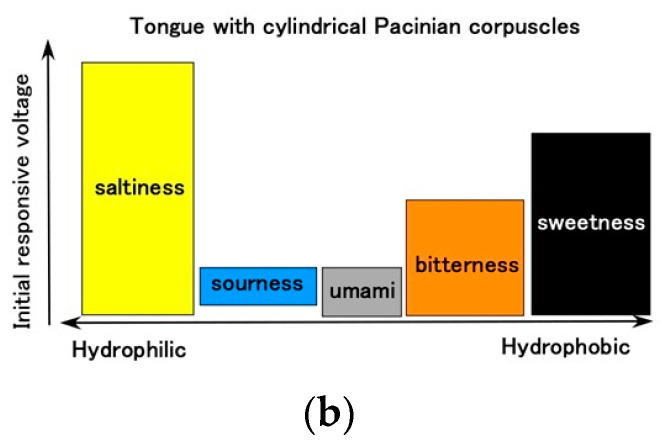
Ultimately arranged relation of initial response voltage to five tastes: (**a**) for layered Pacinian corpuscles; and (**b**) for cylindrical Pacinian corpuscles.

The results ultimately arranged by inducing from the results for ORP, pH, conductivity first described in Figure 8 are presented in Figure 16. The layered type was characterized by the largest response to bitterness, while the cylindrical type responded most to saltiness. These results are in contrast to those of the physicochemical properties of the five basic taste qualities in other reports [28].

The series of cyclic voltammogram plots showing the relation between the electric current *I* and voltage *V* measured by potentiostat (HA-151B, Hokuto Denko Co., Ltd., Tokyo, Japan) at 50-mHz scan rates with the potential domain of −1.5–1.5 V represent significant information, because gustation depends on electrochemical behavior.

**Figure 17:**
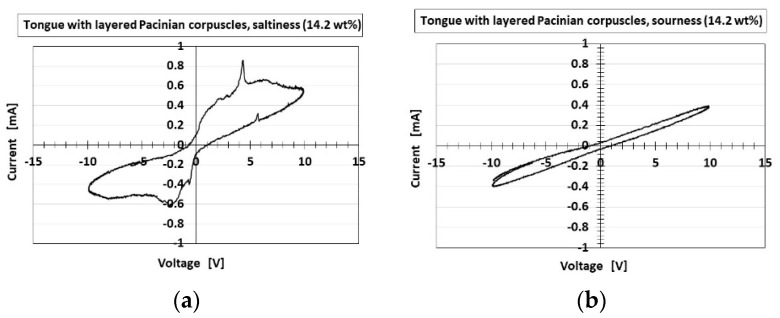

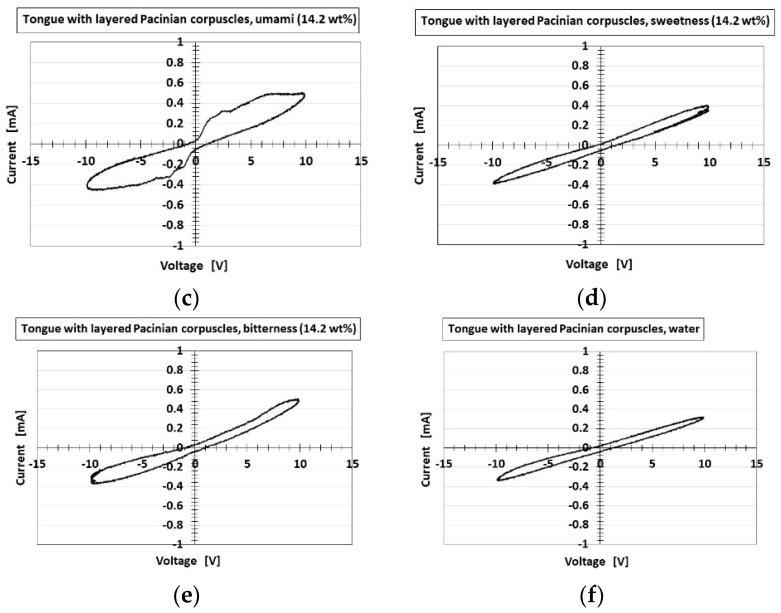
Experimental results of cyclic voltammogram plots for layered Pacinian corpuscles responding to: (**a**) saltiness; (**b**) sourness; (**c**) umami; (**d**) sweetness; (**e**) bitterness; (**f**) water.

**Figure 18:**
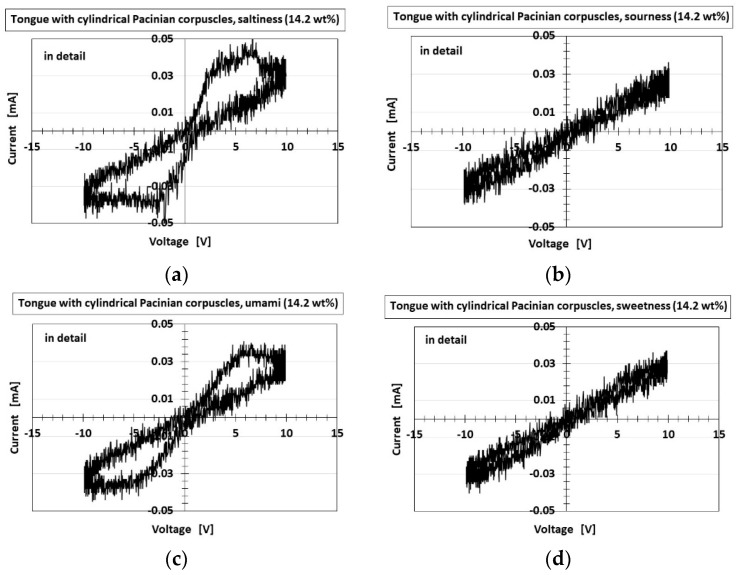

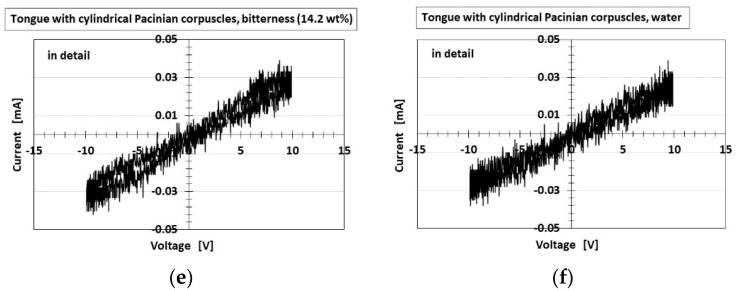
Experimental results of cyclic voltammogram plots for cylindrical Pacinian corpuscles responding to: (**a**) saltiness; (**b**) sourness; (**c**) umami; (**d**) sweetness; (**e**) bitterness; (**f**) water: the right figures are magnified from the left figures.

Figure 17 and Figure 18 present the *I*–*V* characteristics for the layered and cylindrical types, respectively, as well as a comparison to water. The cyclic form differed with the five tastes, allowing the artificial tongue to discriminate between them. Saltiness had the largest cyclic area, followed by umami. Sourness, sweetness, and bitterness had a smaller cyclic area than saltiness and umami. Sourness and bitterness had a slightly larger area at minus *V* than at plus *V*, while the cyclic area of sweetness was the same at plus *V* and minus *V*. The cyclic area of bitterness at plus *V* was slightly larger than that of sourness. This quantitative order of the cyclic area was similar to that of pH, for which the response was in the following order: saltiness, umami, bitterness, sweetness, sourness, as shown in Figure 8. This was because higher [OH^−^] (i.e., less [H^+^]) or lower ORP (i.e., more [red] than [ox]) created a larger cyclic area. These results are the characteristic findings of the HF-rubber made gustatory receptor embedded in the artificial tongue showing that the cyclic form is different by five tastes.

The gustatory case of the cyclic voltammogram provided the same results as that of the initial response voltage and the force and thermal responses, which indicated that the layered type had the larger response and was dependent on the different configuration, as shown in Figure 5 and Figure A2. The cause of the results is due to the fact that the number of the parts of condenser in the case of the layered type is more than that in the case of the cylindrical type, as is the reason in the case of normal force. Namely, more hairs adhered to the condensers to make the cyclic area larger, resulting from the ionized hairs as shown in Figure 6. Furthermore, regarding the cylindrical type, the saltiness and umami had the largest cyclic area in any other tastes. The result and the cause are the same as the ones in the layered type.

## 5. Conclusions

We produced an artificial tongue composed of rubber embedded with gustatory receptors to mimic the human tongue and investigated its gustatory response to five tastes: saltiness, sourness, sweetness, bitterness, and umami. As the human tongue is soft and elastic, we created the artificial tongue by using a soft rubber. In addition, it should be conductive to react to taste by electric signal. By utilizing our proposed HF rubber and electrolytic polymerization technique, a self-powered gustatory receptor could be produced, and an artificial tongue embedded with the receptor. In the case of layered Pacinian corpuscles, the built-in voltage of the artificial tongue was typically responsive not only to normal and shear forces but to thermal variations, more so than the concentric-cylindrical Pacinian corpuscles. The other characteristic findings of the present study were that the initial response voltage, which was specifically arranged, and the cyclic voltammogram form, varied according to the five kinds of tastes. This was due to the ORP, pH, and conductivity. This result again showed that the layered Pacinian corpuscles were more responsive than the cylindrical Pacinian corpuscles.

There were also intriguing structural features of the HF-rubber gustatory receptor by different configurations such that the mechanism between the ionic response to saltiness and sourness, and the G protein responsive process to sweetness, bitterness, and umami were different. In particular, in this study, the results of the distinctive cyclic voltammogram form demonstrated that the five tastes could be differentiated. This could contribute to the development of taste estimation in food and beverage companies, the development of medicine in pharmaceutical industries, and so on.

## Figures and Tables

**Table 1:** Ingredients of HF rubber for the fabrication of the gustatory receptor.

		HF Rubber 1	HF Rubber 2	HF Rubber 3	HF Rubber 4
Ingredients	water	3 g	3 g	1 g	1 g
	Sodium tungstate (VI) dehydrate (Na_2_WO_4_ 2H_2_O, Fujifilm Wako Chemical Co., Ltd., Osaka, Japan)	0.5 g	0.5 g	-	0.5 g
	TiO_2_ (Anataze type, Fujifilm Wako Chemical Co., Ltd., Osaka, Japan)	0.5 g	0.5 g	0.5 g	0.5 g
	HF	1 g	1 g	1 g	1 g
	NR-latex (Ulacol; Rejitex Co., Ltd., Atsugi, Japan)	3 g	3 g	3 g	3 g
	CR-latex (671A; Showa Denko Co., Ltd., Tokyo, Japan)	3 g	3 g	3 g	3 g
	Carbonyl Ni powder (No. 123, Yamaishi Co., Ltd., Noda, Japan)	3 g	3 g	3 g	3 g

## Data Availability

Not applicable.

## Appendix A

The procedure of HF-rubber fabrication for the artificial cylindrical Pacinian is shown in Figure A1, and was described in a previous study of artificial equilibrium and auditory organs [23].

The equivalent electric circuit of the HF-rubber artificial cylindrical Pacinian is shown in Figure A2, which was also used in a previous study of artificial equilibrium and auditory organs [23].
